# Genome-Wide Association Studies Revealed Significant QTLs and Candidate Genes Associated with Backfat and Loin Muscle Area in Pigs Using Imputation-Based Whole Genome Sequencing Data

**DOI:** 10.3390/ani12212911

**Published:** 2022-10-24

**Authors:** Jie Li, Jie Wu, Yunhua Jian, Zhanwei Zhuang, Yibin Qiu, Ruqu Huang, Pengyun Lu, Xiang Guan, Xiaoling Huang, Shaoyun Li, Li Min, Yong Ye

**Affiliations:** 1 Guangdong Guangken Animal Husbandry Group Co., Ltd., Guangzhou 510000, China; 2 College of Veterinary Medicine, South China Agricultural University, Guangzhou 510642, China; 3 National Engineering Research Center for Breeding Swine Industry, College of Animal Science, South China Agricultural University, Guangzhou 510642, China; 4 Zhanjiang State Farm Livestock Co., Ltd., Zhanjiang 524022, China; 5 State Key Laboratory of Livestock and Poultry Breeding, Ministry of Agriculture Key Laboratory of Animal Nutrition and Feed Science in South China, Guangdong Public Laboratory of Animal Breeding and Nutrition, Institute of Animal Science, Guangdong Academy of Agricultural Sciences, Guangzhou 510640, China

**Keywords:** pig, GWAS, genotype imputation, BF, LMA

## Abstract

**Simple Summary:**

Backfat thickness (BF) and loin muscle area (LMA) are important characteristics in pig breeding. The identification of the quantitative trait loci (QTLs) and genes closely associated with these carcass traits may be useful for pig molecular breeding. In this study, genome-wide association studies (GWAS) using imputation-based whole genome sequencing data of four selected phenotypic traits (adjusted 100 kg BF and LMA, adjusted 100 kg BF estimate breeding values and LMA estimate breeding values) were conducted in a total of 1131 pigs from three pure breeds (French Yorkshire, Landrace, and Duroc). As a result, we highlighted *CCND2* and *SHANK2* genes as strong candidates affecting BF traits. The results of this study may provide additional insight into the genetic contribution of these genes to carcass meat production capabilities in pig breeding and enhancing lean meat percentages in pigs.

**Abstract:**

Improvement of carcass features is an essential goal in pig genetic breeding programs. Backfat (BF) and loin muscle area (LMA) are important carcass production metrics and useful indicators of pig production performance and lean meat rate. However, the genetic architecture of BF and LMA traits remains elusive. To identify quantitative trait loci (QTLs) and genes associated with these traits, we performed a genome-wide association study (GWAS) using imputation-based whole genome sequencing data for four phenotypes (adjusted 100 kg BF and LMA, adjusted 100 kg BF EBV and LMA EBV) in 1131 pigs from 3 breeds (French Yorkshire, Landrace, and Duroc). After genotype imputation and quality control, 14,163,315 single nucleotide polymorphisms (SNPs) were retained for further analysis. For the adjusted 100 kg BF trait, using the 2-LOD drop method, a QTL with a 13.4 Kb interval (2.91 to 2.93 Mb on SSC2) and containing a *SHANK2* gene was defined. In addition, two QTLs with 135.40 Kb (from 66.10 to 66.23 Mb) and 3.12 Kb (from 66.886 to 66.889 Mb) intervals containing *CCND2* and *TSPAN11* genes, respectively, were found on SSC5. For the BF-EBV trait, two QTLs (128.77 Kb from 66.10 to 66.23 Mb on SSC5 and 42.10 Kb from 2.89 to 2.93 Mb on SSC2) were identified. Notably, *CCND2* and *SHANK2* were the only candidate genes in their respective QTL interval. Furthermore, we detected a 3.33 Kb (66.106 to 66.110 Mb on SSC2) haplotype block which was detected as affecting the BF_EBV trait, which only contained the *CCND2* gene. Thus, we suggested *CCND2* and *SHANK2* as strong candidate genes for regulating the BF trait for pigs. The empirical confidence intervals of the QTLs were 1.14 Mb (165.65 to 166.79 Mb on SSC6) for adjusted 100 kg LMA and 1.49 Mb (165.26–166.74 Mb on SSC6) for LMA-EBV. These two confidence intervals contained 13 and 28 annotated genes, respectively. Our results provide a deeper understanding of the genetic basis of pig carcass traits. The identified molecular markers will be useful for selecting breeding lines for breeding pigs with superior carcass traits.

## 1. Introduction

The domestic pig (*Sus scrofa*) is widely recognized as an important agricultural livestock species and a crucial biomedical model. Pork has always been a major source of animal protein for people. Therefore, it is essential for pig breeders to concentrate their genetic selection efforts on improving growth rates and lean meat percentages. Backfat (BF) is a crucial carcass parameter in swine production because it influences the pigs’ growth rate, feed efficiency, and reproductive success [1,2]. Another valid metric for determining carcass meat production capability is pigs’ loin muscle area (LMA) which is negatively correlated with BF [3]. Previous studies have shown that LMA has a moderate heritability of 0.46–0.48, compared to moderate to high heritability for the BF trait, and both may be improved through genetic selection processes [4,5]. Therefore, a deeper understanding of pigs’ BF and LMA will help forecast carcass meat production capacity and estimate lean meat percentage [6].

With advances in sequencing technology, multiple genes, quantitative trait loci (QTL), and single nucleotide polymorphisms (SNPs) associated with pig carcass traits have been found. In the pig QTL database, 415 and 594 QTLs associated with LMA and BF were identified, respectively [7]. Many previous studies have found many potential candidate genes associated with the LMA and BF traits of pigs. For example, in commercial female pigs, melanocortin−4 receptor (*MC4R*) and coiled-coil-helix-coiled-coil-helix domain 3 (CHCHD3) were recently identified as candidate genes for LMA, whereas insulin-like growth factor 2 (IGF2) and bone morphogenetic protein (BMP2) were shown to be associated with BF [8]. In a Large White × Minzhu F2 pig resource population, myosin heavy chain 3 (MYH3) and myosin heavy chain 3 (MYH13) may affect the LMA phenotype [9]. In the Italian Large White pig population, pyruvate kinase type M2 (*PKM2*) was shown to be associated with BF [10]. Furthermore, as a widespread tool in genetic association studies, genotype imputation can be used to obtain more genotypes at lower cost by imputing from low to high density SNP markers or even whole-genome sequence (WGS) markers, thus indirectly enabling genome-wide sequence variation to be associated with complex and economically important traits in pig production [11]. In this study, we performed a GWAS using imputation-based whole genome sequencing data to reveal candidate genes associated with BF and LMA traits in 1131 pigs from three breeds (French Yorkshire, Landrace, and Duroc). For multi-breeds genomic analysis, this study aimed to identify novel genomic regions and candidate genes for BF and LMA traits across these breeds. The molecular markers discovered can be used in the genetic improvement of pigs and improve breeding programs.

## 2. Materials and Methods

### 2.1. Animals and Phenotypes

In this study, the 1131 experiment pigs consisted of three pure breeds, including 651 French Yorkshire, 294 Landrace and 186 Duroc. All pigs sustained uniform feeding conditions and were raised on three farms at Guangdong Guangken Group Co., Ltd. (Maoming, China). Phenotypic records of BF and LMA were measured when the pigs were approximately 150 days old. BF and LMA were measured by B-mode ultrasound scanning at a point 5 cm from the dorsal midline at the 12/13th rib. The adjusted 100 kg BF was calculated by the following formula:

adjusted 100 kg BF (mm) = Measured BF + (100 − Measured weight) × Measured BF/(Measured weight − B)(1)

where B is different for sex and breed, and the value is as follows:

male: Yorkshire = −7.277; Landrace = −5.623; Duroc = −6.240(2)

female: Yorkshire = −9.440; Landrace = −3.315; Duroc = −4.481(3)

LMA was adjusted to 100 kg using the formula below:

adjusted 100 kg LMA (cm^2^) = Measured LMA + (100 − Measured weight) × Measured LMA/(Measured LMA + 155) (4)

The phenotypic correlation between the BF and LMA traits was estimated by Pearson’s correlation coefficient.

### 2.2. Breeding Value Predictions

The estimated breeding values (EBV) of the three pig populations for BF and LMA traits were estimated by best linear unbiased prediction (BLUP) [12] using pedigree information. The following mixed linear model was used:

(5) y=Xb+Za+ε

where y is the vector of phenotypes, b is a vector of fixed effects (including sex, 2 levels), a is a vector of the random additive genetic effects of individual animals, ε is the vector of random effects. X and Z  are the incident matrices relating to fixed and random effects, a is distributed as $a\;\sim \;N\left(0,A\sigma^{2}a\right)$, where $\sigma^{2}a$ is the additive genetic variance and A is the additive numerator relationship matrix from pedigree. ε is distributed as $\varepsilon \;\sim \;N\left(0,I\sigma^{2}\varepsilon \right)$, where I is an identity matrix and $\sigma^{2}$ ε is the residual variance. 

### 2.3. Genotype Imputation and Quality Control

Ear samples were collected from each pig and stored in 75% alcohol at −20 °C;. DNA was extracted from each sample and the quality of the DNA was determined using the A260/280 and A260/230 ratios. All animals were genotyped with the China Chip-1 Porcine SNP50 BeadChip (Beijing Compass Agritechnology Co., Ltd., Beijing, China), which contains 51,315 SNP markers across the entire genome. Genotyping was conducted as described by Ding et al. [13]. The SNPs set included 33,887 SNPs for imputation after quality control (QC). After genotyping, we increased the genotype data to whole genome sequence level by imputation strategy. The Swine Imputation (SWIM) Server tool [14] was used to conduct genotyping imputation between target and reference genotype data using default parameter values. Reference haplotype panels were generated from whole genome sequencing data of 2259 pigs, which represented 44 breeds, and the genotype imputation accuracy was of an average concordance rate in excess of 97%, non-reference concordance rate 91%, and r^2^ 0.89. Moreover, the reference population was mainly composed of Landrace (*n* = 651), Yorkshire (*n* = 543), and Duroc (*n* = 485) breeds. Thus, we submitted chip data from a total of 1131 pigs of three breeds in this study to SWIM for imputation together. The QC of imputation-based whole genome sequence data was carried out with PLINK [15] software v1.90 using the following parameters: Individuals with an overall call rate of less than 95% were excluded; SNPs with call rates less than 90%, minor allele frequency lower than 0.05, and Hardy–Weinberg *p*-value greater than 10^−6^ were ruled out. Only SNPs located on the autosome chromosomes remained in this study. After QC, 1131 pigs and 14,163,315 SNPs remained for subsequent analyses. GCTA [16] was used to estimate genetic correlation in the bivariate mode and was also used to compute the genomic heritability.

### 2.4. Genome-Wide Association Study

Imputation-based GWAS for the four traits were conducted using GEMMA software v0.98.1 [17]. The following was the statistical linear mixed model:

(6) $$y=W\alpha +X\beta +u+\varepsilon ;\;u\;\sim \;MVN_{n}\;\;\left(0,\lambda \tau^{-1}K\right),\;\varepsilon \;\sim \;MVN_{n}\;\;\left(0,\;\tau^{-1}I_{n}\right)$$

where y refers to a vector of the phenotypes for all animals; W is the incidence matrix of fixed effects including sex effects and the top five principal components calculated by GCTA; α represents the vector of corresponding coefficients including the intercept; X is the vector of SNP genotypes; β is the corresponding effect of the marker; u is the vector of random effects; ε is the vector of random residuals; both u and ε follow the multivariate normal distribution; λ is the ratio of two variance components; $\tau^{-1}$ is the variance of the residual errors; K is a standardized relatedness matrix estimated by GEMMA software; $I_{n}$ is an identity matrix, and *n* is the number of animals. We used a genome-wide significant threshold of *p* = 5 × 10^−8^ to declare the significance as described in previous study [18]. As described in previous studies, the QTL intervals were determined by a 2-LOD drop method. One unit of −log (*p*-value) was roughly equivalent to one unit of logarithm of the odds (LOD) value and all SNPs in each SSC with a LOD-score higher than the peak LOD-score (−log *p*-value) minus 2 were retained [19]. 

### 2.5. Haplotype Block Analysis and Candidate Gene Identification

The PLINK v1.90 and LDBlockShow v1.40 [20] were used to perform haplotype block analysis for chromosomal regions with multiple significantly clustered around the top SNP to evaluate the linkage disequilibrium (LD) pattern of the region. The functional genes were searched based on *Sscrofa* 11.1 genome version (http://asia.ensembl.org/Sus_scrofa/Info/Index, accessed on 1 May 2022). Candidate genes were selected in the candidate QTL region based on the 2-LOD drop method and haplotype block analysis. Furthermore, we manually queried the literature for the information about the associations between all candidate genes and the analyzed traits in this study.

## 3. Results

### 3.1. SNP Genotyping and Phenotypic Variation

**Figure 1:**
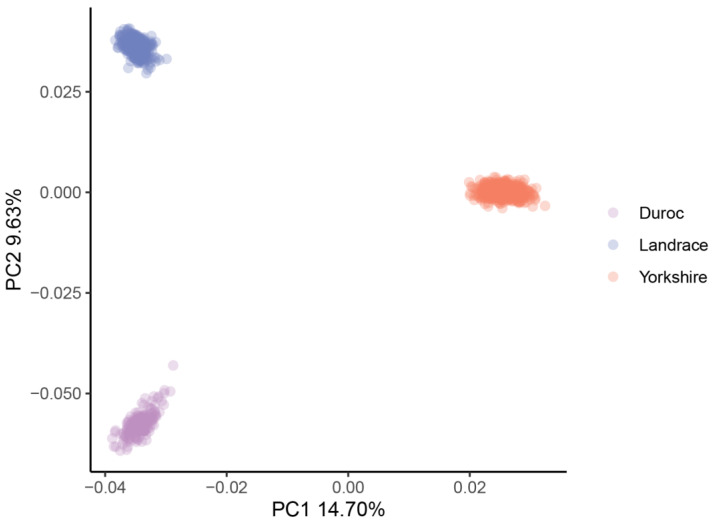
PCA plot for the three pig populations. PC1 as first principal component; PC2 as second principal component.

Table 1 contains a summary of the phenotypic data (adjusted 100 kg BF, adjusted 100 kg BF EBV, adjusted 100 kg LMA and adjusted 100 kg LMA EBV) of the 1131 pigs. A negative genetic correlation was found between adjusted 100 kg BF and LMA (−0.33) and between adjusted 100 kg BF and LMA EBV (−0.08). Genotyping imputation yielded a genotype density of 30,489,782 SNPs. After QC, 14,163,315 SNPs were retained for subsequent analysis in this study. Figure 1 shows a scatterplot based on the first two principal components in principal component analysis.

### 3.2. GWAS for BF Trait

**Figure 2:**
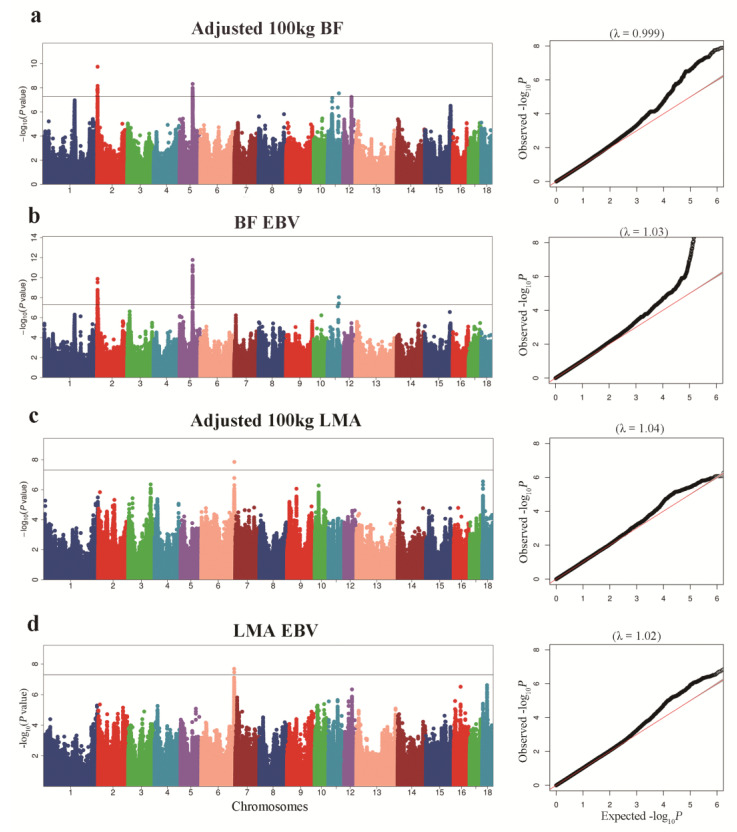
Manhattan plots of GWAS for BF and LMA traits in pigs. In the Manhattan plot, the solid represent a significance threshold of 5 × 10^−8^. Manhattan plot and QQ plot for: (**a**) adjusted 100 kg BF, (**b**) adjusted 100 kg BF EBV; (**c**) adjusted 100 kg LMA; (**d**) adjusted 100 kg LMA EBV.

Manhattan plots of GWAS for adjusted 100 kg BF and BF-EBV traits and the corresponding Q–Q plots are shown in Figure 2a,b. Significant SNPs underlying the QTLs as detected through the association study for BF trait are shown in Table 2 and Table S1. We detected three QTLs significantly associated with adjusted 100 kg BF trait. The QTL on SSC2 had an empirical confidence interval of 13.41 Kb, which encompassed one annotated gene, SH3 and multiple ankyrin repeat domain 2 (*SHANK2*). We identified two QTLs on SSC5 with intervals of 135.40 Kb and 3.11 Kb. Cyclin D2 (*CCND2*) and Tetraspanin 11 (*TSPAN11*) were identified as candidate genes associated with these QTLs. For the BF-EBV trait, we detected one QTL on SSC5 with an interval of 128.77 Kb, and mined *CCND2* as the candidate gene associated with it. Gene *SHANK2* was also identified in a QTL with an interval of 42.10 Kb on SSC2 for BF-EBV trait. 

**Figure 3:**
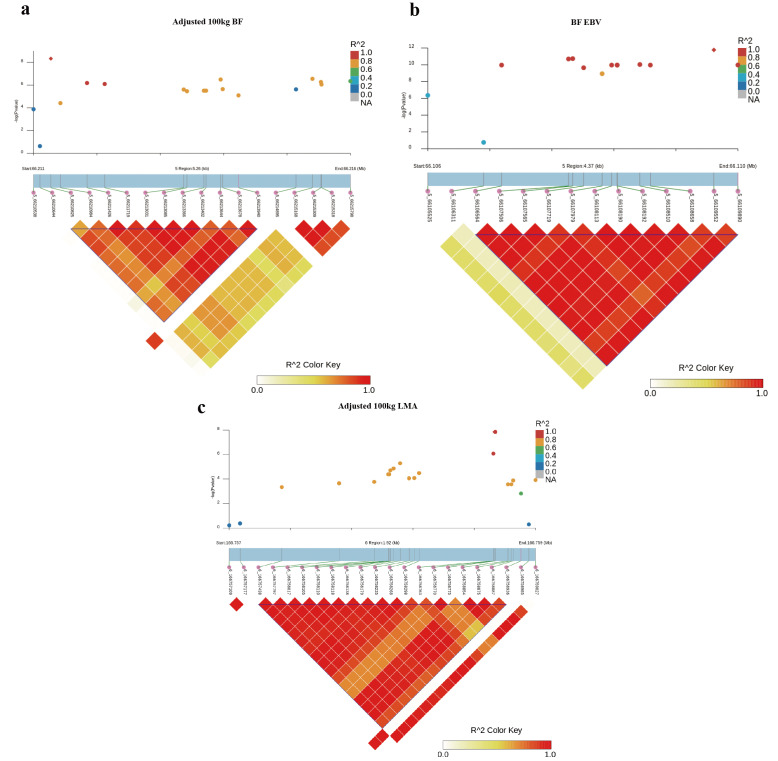
Haplotype block analysis of Top SNPs associated with BF and LMA traits. LD blocks are marked with triangles. LD (r^2^) value between two SNPs were indicated by the colors of the squares. Diamond squares in the scatter plot indicate the most significant SNPs associated with traits. Scatter plot showing the LD between other SNPs and the most significant SNPs: (**a**) adjusted 100 kg BF; (**b**) adjusted 100 kg BF EBV; (**c**) adjusted 100 kg LMA EBV.

Furthermore, LD analysis around the most significant SNP for adjusted 100 kg BF trait on SSC5 identified one haplotype block of 3.10 Kb between 5_66210825 and 5_66213940 SNPs (Figure 3a). In addition, another haplotype block of 3.33 kb between 5_66106564 and 5_66109890 SNPs consisting of 14 SNPs associated with BF-EBV trait was detected (Figure 3b). Of these, 10 SNPs were significantly associated with BF_EBV trait and only contained a *CCND2* gene.

### 3.3. GWAS for LMA Trait

For adjusted 100 kg LMA and LMA-EBV traits, we identified one QTL on SSC6 that surpassed the significant threshold. The empirical confidence intervals of the QTL were 1.14 Mb (165.65–166.79 Mb) for adjusted 100 kg LMA and 1.49 Mb (165.26–166.74 Mb) for LMA-EBV (Table 3 and Figure 2c,d). These two confidence intervals contained 13 and 28 annotated genes, respectively. In addition, we detected an LD block of 1.50 Kb between 6_166757438 and 6_166758936 SNPs, which contained two significant SNPs for LMA-EBV trait (Figure 3c). *RNF220* was the only gene located in this block.

## 4. Discussion

BF and LMA traits are both complex quantitative traits regulated by a main effect gene and a set of micro effect genes in pigs [21,22]. With the rapid advance in genome sequencing, many researchers have employed high-density microarrays for GWAS analysis to profoundly improve economically important features in pigs by applying molecular markers in selection. However, because most research is focused on a particular breed, the use of these molecular markers across breeds is limited [23]. In this study, we performed GWAS analysis of BF and LMA traits in 1131 pigs from three breeds (French Yorkshire, Landrace, and Duroc) and greatly increased the SNPs available for GWAS analysis by genotype imputation. To adjust for possible population stratification, we performed PCA and used the top five principal components as covariates in statistical analyses. In the QQ plot (Figure 2), the genomic inflation factor (λ) of GWAS ranged from 0.99 to 1.05, indicating that we properly adjusted the effect of population stratification in GWAS.

Genotype imputation can be used to infer genotypes at untyped loci, thus playing an important role in current genome-wide association studies [24]. In this study, we increased the number of usable SNP loci by genotype imputation to obtain more reliable GWAS results. After imputation, for BF traits, *CCND2* and *SHANK2* genes were the only genes in the respective QTL support interval (a drop in LOD score of 2). Most importantly, *CCND2* was the only gene in the LD block of the SNPs associated with the BF EBV trait. Therefore, *CCND2* and *SHANK2* may be considered strong candidate genes as quantitative trait gene (QTG) for pigs’ BF trait. *CCND2* is involved in the growth of pancreatic islets, which regulate animal growth through its hormones [25]. In a GWAS investigation on Danish Landrace, Large White, and Duroc pigs, Le et al. found that the *CCND2* gene may be related to the growth performance of pigs and is as a possible candidate gene for backfat conformation in Landrace [25]. In addition, in a related study using mice models, *CCND2* was significantly associated with animal body weight and total body fat amount [26]. Our results support these observations. *SHANK2*, a member of the Shank protein family, was found to be associated with childhood obesity, and to have an influence on estradiol blood concentrations [27,28]. In a study by Clemens et al., the *SHANK2* gene was identified as likely to affect the meat to fat ratio in pigs [29]. However, no signals were detected in individual breed GWAS results (Figures S1–S3).

In our study, a total of 23 candidate genes associated with LMA traits were detected in the region spanning from 165.65 to 166.74 Mb on SSC6. In particular, a study by Ma et al. showed that ring finger protein 220 (*RNF220*) (+/−) mice exhibited different typical amyotrophic lateral sclerosis (ALS) pathological features [30]. In our study, *RNF220* was the only gene in the LD block of the associated with pigs’ LMA trait. Therefore, we hypothesize that the *RNF220* gene has an effect on the LMA in pigs and thus warrants further study. Peroxiredoxin 1 (*PRDX1*), a member of the peroxiredoxin family of antioxidant enzymes, regulates with hematocrit levels and hemoglobin concentration. As a candidate gene associated with the immune health in pigs, *PRDX1* may indirectly affect the growth and LMA traits of pigs [31]. However, no definite link has been established between the function of these genes and the LMA trait, and, therefore, more research is required to determine this relationship.

## 5. Conclusions

In this study, we conducted imputation-based GWAS for BF and LMA traits to detect significant QTLs and genes in large pig populations consisting of three breeds. Significant QTLs and genes were identified as being associated with the traits analyzed in pigs. We highlighted *CCND2* and *SHANK2* as strong candidate genes affecting BF trait. These results will advance our understanding of the genetic basis of complex traits in pigs. In addition, the identified SNPs will be useful for the genetic improvement of BF and LMA traits in pigs by exploiting associated SNPs in genomic selection.

## Figures and Tables

**Table 1:** Phenotype and heritability statistics for BF and LMA in three pig populations.

Traits	Mean (±sd)	C.V.	*h^2^*	Phenotypic Correlation	Genetic Correlation
Adjusted 100 kg BF	11.38 ± 2.82	24.78	0.56 ± 0.05	−0.23	−0.33 ± 0.09
Adjusted 100 kg LMA	37.22 ± 4.75	12.76	0.50 ± 0.05		
BF EBV	0.05 ± 0.74	14.80			
LMA EBV	0.04 ± 1.90	47.50			

(1) Adjusted 100 kg backfat, adjusted 100 kg loin eye area, adjusted 100 kg backfat EBV and adjusted 100 kg loin eye area EBV. (2) Mean (standard deviation). (3) Coefficient of variation. (4) Heritability (standard error). (5, 6) Phenotypic and genetic correlations (standard deviation) of BF and LEA traits value. All of the phenotypic correlation coefficients are significant with *p*-value < 0.05.

**Table 2:** Top 10 significant SNPs and genes associated with BF traits in the QTL intervals identified by 2-LOD drop method.

Traits	SSC	Positoin	*p*-Value	Consequence	Candidate Gene
Adjusted 100 kg BF	5	66210825	4.73 × 10^−9^	intergenic_variant	*CCND2, TSPAN11*
	5	66109552	1.02 × 10^−8^	splice polypyrimidine tract variant, splice region variant, intron variant	
	5	66225671	1.23 × 10^−8^	intergenic_variant	
	5	66231984	1.78 × 10^−8^	intergenic_variant	
	5	66231994	2.31 × 10^−8^	intergenic_variant	
	5	66231774	2.61 × 10^−8^	intergenic_variant	
	5	66231974	3.28 × 10^−8^	intergenic_variant	
	5	66232303	3.38 × 10^−8^	intergenic_variant	
	5	66223267	3.72 × 10^−8^	intergenic_variant	
	5	66224507	4.03 × 10^−8^	intergenic_variant	
	2	2915752	1.76 × 10^−10^	intron_variant	*SHANK2*
	2	2927340	6.84 × 10^−9^	intron_variant	
	2	2924189	1.08 × 10^−8^	intron_variant	
	2	2913910	1.27 × 10^−8^	intron_variant	
	2	2913928	1.27 × 10^−8^	intron_variant	
	2	2913943	1.27 × 10^−8^	intron_variant	
	2	2914889	1.27 × 10^−8^	intron_variant	
	2	2915433	1.27 × 10^−8^	intron_variant	
	2	1521939	1.37 × 10^−8^	intergenic_variant	
	2	2919066	1.50 × 10^−8^	intron_variant	
BF EBV	5	66109552	1.70 × 10^−12^	splice polypyrimidine tract variant, splice region variant, intron variant	*CCND2*
	5	66230535	5.98 × 10^−12^	intergenic_variant	
	5	66227438	6.17 × 10^−12^	intergenic_variant	
	5	66103958	6.77 × 10^−12^	intron_variant	
	5	66210825	1.24 × 10^−11^	intergenic_variant	
	5	66220545	1.27 × 10^−11^	intergenic_variant	
	5	66224773	1.64 × 10^−11^	intergenic_variant	
	5	66224775	1.64 × 10^−11^	intergenic_variant	
	5	66224782	1.64 × 10^−11^	intergenic_variant	
	5	66230608	1.70 × 10^−11^	intergenic_variant	
	2	2915752	1.35 × 10^−10^	intron_variant	*SHANK2*
	2	2927340	2.99 × 10^−10^	intron_variant	
	2	2919084	1.71 × 10^−9^	intron_variant	
	2	2919066	2.07 × 10^−9^	intron_variant	
	2	2928547	2.13 × 10^−9^	intron_variant	
	2	2927984	3.34 × 10^−9^	intron_variant	
	2	2924104	5.16 × 10^−9^	intron_variant	
	2	2924106	5.16 × 10^−9^	intron_variant	
	2	2927278	7.83 × 10^−9^	intron_variant	
	2	2928297	9.32 × 10^−9^	intron_variant	

(1) Adjusted 100 kg backfat and adjusted 100 kg backfat EBV. (2) Sus scrofa chromosome. (3) SNP positions in Ensembl. (4) *p*-value. (5) The consequence of detected variants was determined using Ensembls Variant Effect Predictor (VEP). (6) Candidate gene.

**Table 3:** Significant SNPs and genes associated with LMA traits in the QTL intervals identified by 2-LOD drop method.

Traits	SSC	Position	*p*-Value	Consequence	Candidate Gene
Adjusted 100 kg LMA	6	166758770	1.41^−8^	intron_variant	*GPBP1L1, IPP, TMEM69, NASP, PRDX1, TESK2, MMACHC, ARMH1, ENSSSCG00000021448, ENSSSCG00000046843*
	6	166758775	1.41^−8^	intron_variant	
	6	166603876	1.67^−7^	intron_variant	
	6	166786086	5.03^−7^	intron_variant	
	6	166758763	8.36^−7^	intron_variant	
	6	165650769	1.13^−6^	intergenic_variant	
	6	165953286	1.24^−6^	intron_variant	
	6	166744870	1.34^−6^	intron_variant	
LMA EBV	6	165987408	2.06^−8^	intron_variant	*MAST2, PLK3, AKR1A1, UROD, EIF2B3, HECTD3, BEST4, GPBP1L1, IPP, TMEM69, NASP, PRDX1, TESK2, ZSWIM5, DYNLT4, BTBD19, RPS8, MMACHC, ARMH1, ENSSSCG00000021448, ENSSSCG00000046843, ENSSSCG0000003909, ENSSSCG00000035907*
	6	166603876	3.45^−8^	intron_variant	
	6	165924110	7.65^−8^	intron_variant	
	6	165924144	7.65^−8^	intron_variant	
	6	165927020	7.83^−8^	intron_variant	
	6	166000906	1.06^−7^	missense variant, upstream gene variant, downstream gene variant	
	6	166017041	1.35^−7^	upstream gene variant, downstream gene variant	
	6	166629589	1.40^−7^	intron_variant	
	6	166634843	1.40^−7^	intron_variant	
	6	166637452	1.54^−7^	intron_variant	

(1) Adjusted 100 kg loin eye area and adjusted 100 kg loin eye area EBV. (2) Sus scrofa chromosome. (3) SNP positions in Ensembl. (4) *p*-value. (5) The consequence of detected variants was determined using Ensembl Variant Effect Predictor (VEP). (6) Candidate gene.

## Data Availability

Data will be available upon request.

## Supplementary Materials

The following supporting information can be downloaded from: https://www.mdpi.com/article/10.3390/ani12212911/s1, Table S1: Significant SNPs associated with BF and LMA traits in the QTL intervals identified by 2-LOD drop method. Figure S1: Manhattan plot of GWAS based on imputed data for BF and LMA traits in Yorkshire. Figure S2: Manhattan plot of GWAS based on imputed data for BF and LMA traits in Landrace. Figure S3: Manhattan plot of GWAS based on imputed data for BF and LMA traits in Duroc.
