# Supplementary material for: Genome-Wide Association Studies Revealed Significant QTLs and Candidate Genes Associated with Backfat and Loin Muscle Area in Pigs Using Imputation-Based Whole Genome Sequencing Data
Source: Animals (Basel). 2022 Oct 24;12(21):2911. doi: 10.3390/ani12212911 (PMC9655224; doi:10.3390/ani12212911)
Supplement: Supplementary file 1 [file animals-12-02911-s001.zip › animals-1916802-supplementary/animals-1916802-supplementary.pdf]

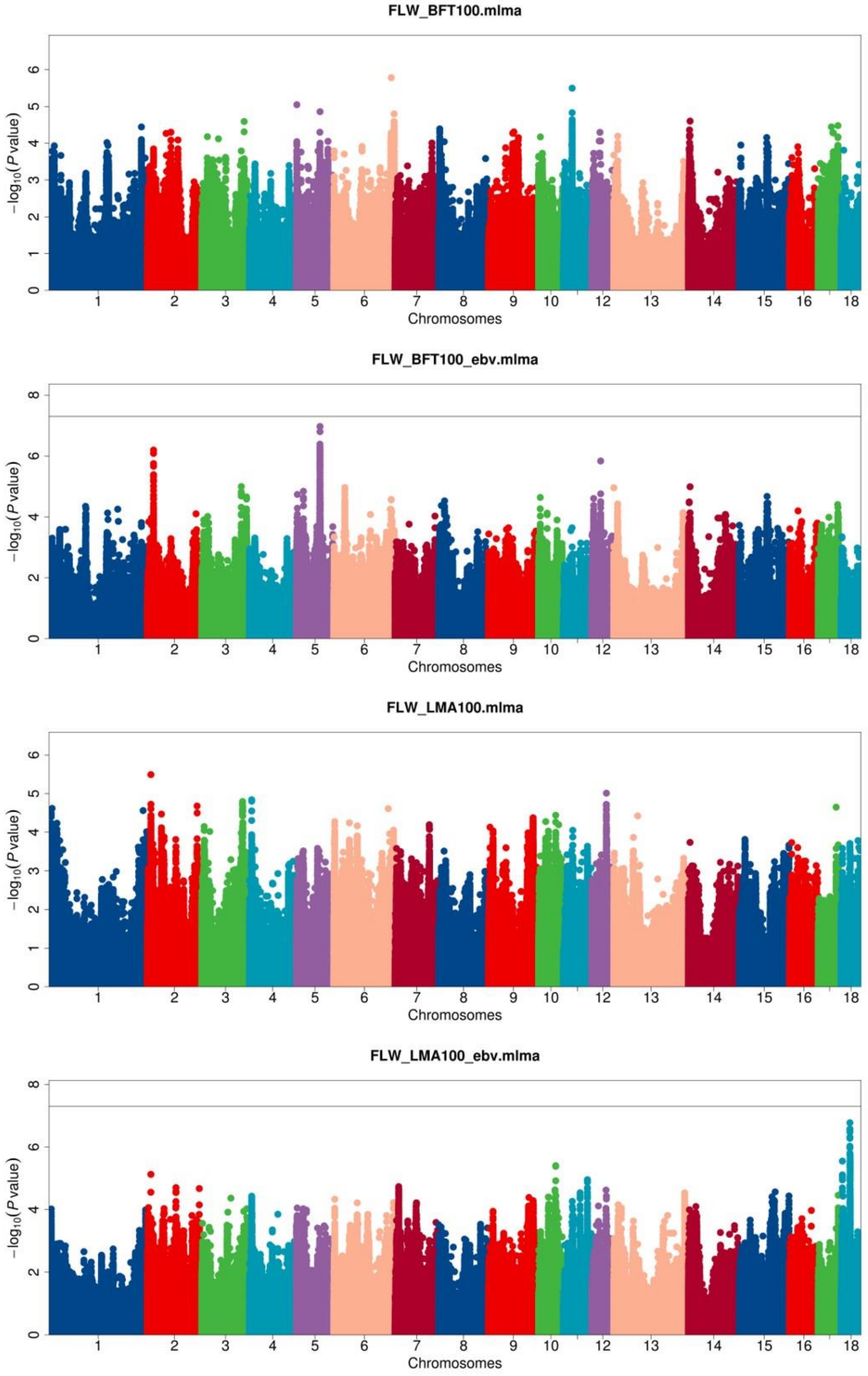

Figure S1 Manhattan plot of GWAS based on imputed data for BF and LMA traits in Yorkshire.

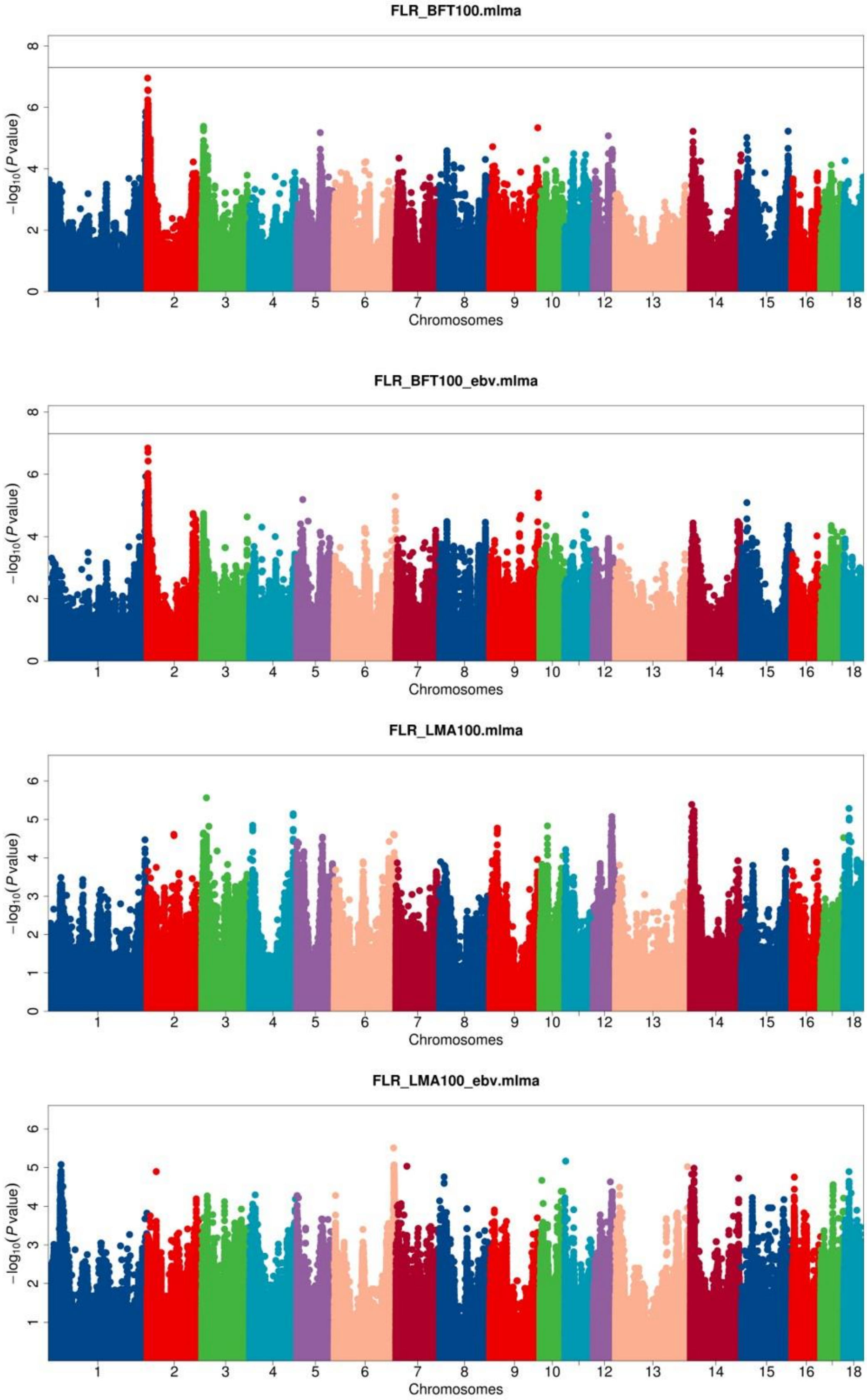

Figure S2 Manhattan plot of GWAS based on imputed data for BF and LMA traits in Landrace.

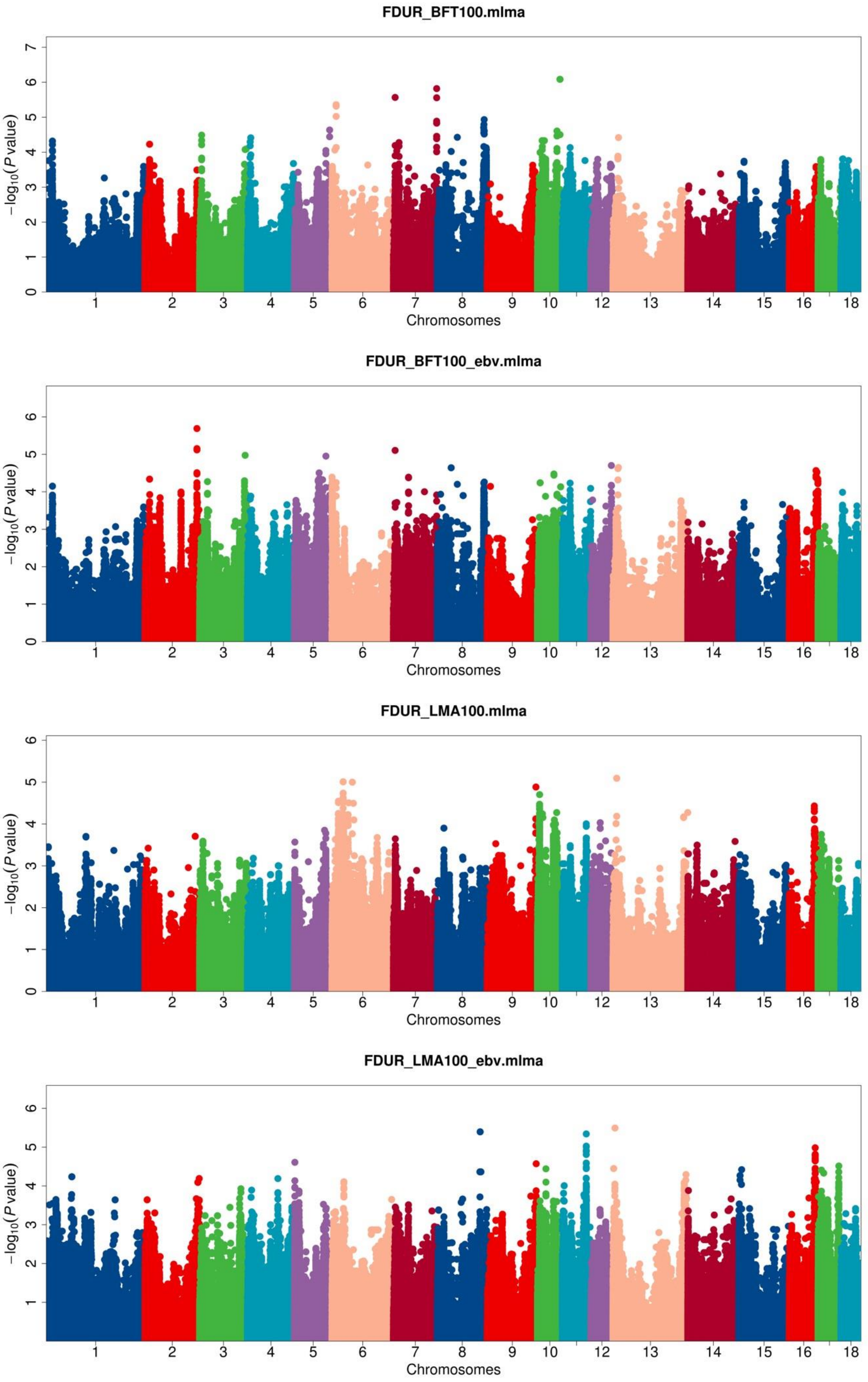

Figure S3 Manhattan plot of GWAS based on imputed data for BF and LMA traits in Duroc.
